# Burnout, Compassion Fatigue, and Compassion Satisfaction Interventions via Mobile Applications: A Systematic Review and a Meta‐Analysis

**DOI:** 10.1111/wvn.70033

**Published:** 2025-05-13

**Authors:** Denis Deriglazov, Júlia Halamová, Lívia Kernová

**Affiliations:** ^1^ Institute of Applied Psychology, Faculty of Social and Economic Sciences Comenius University in Bratislava Bratislava Slovakia

**Keywords:** burnout, compassion fatigue, compassion satisfaction, meta‐analysis, mobile application, systematic review

## Abstract

**Background:**

The increasing prevalence of burnout, compassion fatigue, and reduced compassion satisfaction among healthcare professionals has highlighted the need for effective interventions. Mobile applications offer a promising solution due to their accessibility and low cost.

**Methods:**

This systematic review and meta‐analysis evaluates the effectiveness of mobile interventions in addressing burnout, compassion fatigue, and compassion satisfaction among healthcare professionals, while analyzing subcomponents of burnout to account for the frequently overlapping definitions and symptoms shared by these conditions. We included randomized controlled trials (RCTs) and quasi‐experimental studies published between 2010 and 2024. Data were synthesized using a random effects model, with effect sizes estimated in Hedge's *g*.

**Results:**

Fourteen studies met the inclusion criteria, comprising 11 RCTs and 3 quasi‐experimental studies, with participant numbers ranging from 20 to 2182. Most intervention content focused on mindfulness and meditation (*n* = 7) and resilience‐based programs (*n* = 3). The systematic review indicated mixed results for mindfulness and resilience apps, while most studies that used meditation showed improvements in burnout. Although interventions directly targeting compassion fatigue and compassion satisfaction showed no significant effects, the meta‐analysis revealed improvements in burnout domains, including a significant effect on personal accomplishment (Hedge's *g* = 0.51) and mixed findings for emotional exhaustion. While these interventions do not directly reduce compassion fatigue or raise compassion satisfaction, they may contribute to job satisfaction and a sense of professional efficacy. A sensitivity analysis improved homogeneity, leading to significant effects on emotional exhaustion and the generalizability of our findings.

**Linking Evidence to Action:**

Interventions focused on mindfulness, resilience training, and other strategies via mobile applications enhance personal accomplishment among healthcare professionals and show promising results in reducing emotional exhaustion. Their effectiveness in reducing compassion fatigue, depersonalization, and increasing compassion satisfaction remains inconsistent. Current research predominantly focuses on healthcare professionals, despite evidence suggesting that a broader range of healthcare professionals also suffer from compassion fatigue and burnout. The limited data on compassion fatigue and satisfaction highlights a gap in the current literature, showing the need for further high‐quality studies in the form of RCTs.

## 1 Introduction

The lives of healthcare professionals are marked by daily encounters with suffering, the trauma of others, and work stressors. This constant exposure, together with acute stress and exhaustion, places them at heightened risk of burnout and/or compassion fatigue, impacting their mental health and job performance and reducing patient satisfaction and productivity (Kase et al. 2021).

Burnout arises from chronic workplace stress and is characterized by emotional exhaustion, depersonalization, and a reduced sense of personal accomplishment (Maslach and Jackson 1981). Compassion fatigue, a related but distinct phenomenon, refers to feelings of social, psychological, and biological exhaustion that occur after long exposure to compassionate stress (Wynn 2020).

Although burnout and compassion fatigue can occur simultaneously, the latter specifically includes most of the elements of burnout due to the emotional toll of caring for trauma victims (Forrest et al. 2020). In contrast, burnout includes stressors like excessive workload and lack of support (Cavanagh et al. 2019). However, the conceptual ambiguity surrounding these constructs may undermine both research and clinical efforts to address them effectively. Boscarino et al. (2004) argue that compassion fatigue, burnout, vicarious trauma, and general psychological distress are often conflated in the literature and therefore lack clarity. This lack of conceptual clarity is further explored by Van Mol et al. (2015), who highlight inconsistent definitions of burnout and compassion fatigue, such as whether they are framed as psychological syndromes, occupational hazards, or trauma‐specific outcomes. Such ambiguity may result in insufficient evidence and could lead to poorly designed and ineffective interventions, highlighting the need to evaluate interventions targeting burnout and those addressing compassion fatigue.

Healthcare professionals are particularly vulnerable to compassion fatigue and burnout. Over 50% of physicians experience symptoms of burnout, a significantly higher rate than in other professions (Shanafelt et al. 2015). Other studies indicate that nurses have the highest levels of compassion fatigue, with rates reaching 80%, compared to 59% among general healthcare professionals (Kabunga et al. 2024). The persistent emotional strain associated with being compassionate towards trauma victims can lead to compassion fatigue, which, in turn, can exacerbate burnout by depleting the emotional resources needed for effective caregiving (Figley 1995; Maslach et al. 2001).

In contrast, compassion satisfaction represents the positive feelings derived from the ability to help others and the gratification from competent caregiving (Stamm 2010). Compassion satisfaction is driven by altruistic behavior and the sense of accomplishment, meaningfulness, fulfillment, and satisfaction healthcare professionals feel having improved the well‐being of patients, and it is also known to be a motivator of commitment to the profession (Chen et al. 2022; Harr et al. 2014).

Despite its importance, research highlights a significant gap in equipping staff and professionals with effective measures to mitigate the negative effects of compassion fatigue (Harr et al. 2014). Most social work educational programs rarely address issues related to compassion fatigue, though incorporating preventive information into the curriculum has been suggested as a potential solution. Frameworks such as ProQual could be used to integrate relevant compassion fatigue content into educational courses, with further investigation to assess their efficacy (Van De Mortel et al. 2023).

Research also shows that secondary traumatic stress (67%) and burnout (63%) were found to dominate over compassion satisfaction (23%) (Algamdi 2022). This imbalance and prevalence of compassion fatigue highlight a need for accessible, cost‐effective interventions. In recent years, particularly following the COVID‐19 pandemic, mobile interventions have emerged as significant tools for mitigating the negative impacts of mental distress, including compassion fatigue and burnout (Moore et al. 2024). They offer features such as cognitive behavioral therapy, stress inoculation therapy, mindfulness‐based interventions, stress and time management, psychoeducation, and yoga, which can help professionals manage stress, develop resilience, and enhance well‐being.

Research has shown mobile health applications are effective in alleviating symptoms of depression, anxiety, and improving general well‐being (Lattie et al. 2019). Such interventions delivered through mobile and web platforms can provide essential mental health support, overcoming barriers like the stigma and time constraints associated with traditional mental health services. However, many of the mental health apps available to consumers lack evidence‐based content, which could be harmful (Larsen et al. 2019). While digital tools aimed at mitigating mental distress have been increasingly studied, compassion satisfaction has been less frequently explored in the context of mobile interventions (Moore et al. 2024). Recent findings suggest that mindfulness‐based interventions have been able to improve compassion satisfaction with a small effect size, but studies focusing exclusively on mobile applications have not been synthesized in a meta‐analysis (Li et al. 2025).

Despite the growing importance of mobile interventions in alleviating the distress of healthcare professionals, there is a limited number of current systematic reviews and meta‐analyses on this topic and on compassion fatigue, compassion satisfaction, and burnout. Cavanagh et al. (2019) and Kabunga et al. (2024) investigated the prevalence of compassion fatigue and burnout among healthcare providers. Pospos et al. (2017) explored the efficacy of web and mobile applications in alleviating burnout, depression, and suicide rates. A meta‐review synthesizing findings from seven meta‐analyses indicated that mobile apps might be effective in managing anxiety and depression (Lecomte et al. 2020). However, there is also an emphasized need for more high‐quality studies targeting other mental health issues and specific populations. Ilola et al. (2024) examined the use of mobile applications and online programs specifically focused on mindfulness, self‐compassion, and meditation among workers, but noted the limitations of their study in a relatively small set of studies included in the meta‐analysis and small sample sizes.

These studies reveal the importance of addressing compassion fatigue and burnout and show promising results in mobile app usage among healthcare providers. However, they exhibit several limitations. Many are narrowly focused, often limited to specific interventions like mindfulness (Ilola et al. 2024) or to particular populations such as healthcare providers (Cavanagh et al. 2019; Kabunga et al. 2024). There is also a problem with heterogeneity in the reported data, which can introduce bias, and the methodological quality of the studies (Lecomte et al. 2020), with more rigorous RCTs needed to confirm efficacy. We aim to address the research gap by investigating the effectiveness of mobile interventions on burnout, compassion fatigue, and compassion satisfaction. We focus on the strongest comparison strength for interventions (Cumpston et al. 2019) involving mobile applications using RCT and quasi‐experimental designs.

## 2 Methods

### 2.1 Design

The current study is a systematic review and meta‐analysis focusing on burnout, compassion fatigue, and compassion satisfaction interventions provided through mobile applications for healthcare professionals and aims to analyze their effectiveness.

We followed PRISMA (Preferred Reporting Items for Systematic Reviews and Meta‐Analyses) guidelines to improve the quality of the data and the findings of the review (Page et al. 2021). PRISMA is an evidence‐based set of items that should be reported in systematic reviews and RCT meta‐analyses, particularly for interventions.

### 2.2 Eligibility Criteria

We used the PICO framework (Population, Interventions, Comparison, Outcome) to formulate the research question (Schardt et al. 2007): how effective are mobile app interventions aimed at tackling burnout, compassion fatigue, and compassion satisfaction in healthcare professionals? We selected articles published between 2010 and 2024.

One of the main inclusion criteria was working as a helping professional (for the list of healthcare professionals, refer to the Appendix 1). We use a broad definition of healthcare professionals given by Westergaard (2017), who identified key similarities among practitioners using helping skills in working with people, rather than simply a “therapeutic” definition. The full eligibility criteria are described in Table 1.

**TABLE 1:** Eligibility criteria.

	Inclusion criteria	Exclusion criteria
Population	Helping professionals	Studies with undergraduate students (BSc) who are not employed in any of the professions on the list
Interventions	Any type of mobile‐app based intervention focusing on BO, CF, and CS	Other online and mixed design interventions including face‐to‐face sessions
Comparison	Waitlist control group or an active control group receiving any alternative treatment for BO, CF or CS or any other control condition	Studies with only pre‐post measurements or pre‐post‐follow up measurements
Outcome	BO, CF, and CS	Studies not reporting any of the key measurements
Study Designs	RCT or Quasi‐experimental	Any other type of study
Language	English	Other languages

Abbreviations: BO, burnout; CF, compassion fatigue; CS, compassion satisfaction; RCT, randomized controlled trials.

### 2.3 Search Strategies

A systematic search of SCOPUS (*n* = 159) and Web of Science (WoS) (*n* = 103) identified 262 articles. The search strings enquiry encompassed four categories: “Randomised Controlled Trial,” “Helping professionals,” “Mobile applications,” and “Compassion Fatigue or Compassion satisfaction or Burnout,” with a variety of different subcategories. The main search was performed in November 2023 and updated to include January 30, 2024. The search string for WoS was as follows:

Random* OR “randomized control trial” OR “randomised control trial” OR “randomised control study” or “randomized control study” OR Experiment* Or intervention OR treatment* OR prevention OR prevent* OR beforeafter OR “before after” OR “before‐and‐after” OR Pretest OR “pre test posttest” OR “Pre‐test” OR pretest OR “pre test” OR “post test” OR “Quasi experiment” OR “quasi‐experiment” OR “Post‐test” OR “posttest” OR “post‐test” OR “Control trial” OR “Clinic trial” OR “Follow up” OR “follow‐up” OR Followup OR “follow‐up” OR Longitudinal OR Psychoeducation **(Topic) and** “Mobile Application” OR smartphone OR app or “smartphone technology” or “smartphone app” or “mobile app” or “mobile phone app” or “mobile device app” or “portable software app” or “tablet app” or “digital app” or “MH app” or mhapp OR “mental health app” or Mapp or “M app” or “M health” or “web application” or “digital intervention” or “digital treatment” or dmhi or “digital mental health intervention” OR “e‐learning” OR “online training” **(Topic) and** “Compassion fatigue” or “secondary traumatic stress” or “Secondary trauma” or “vicarious trauma” or Burnout or “work burnout” or “burn out” OR “Compassion satisfaction” **(Topic) and** Nurs* or Doctor* or Psychotherapist* or therapist* or Psychologist* or “Health professional” or professional* or lawyer* or “police officer” or policeman or pharmacist* or nun* or monk* or trainer* or coach* or mentor* or dentist* or Paramedic* or “Homecare worker” or “social worker” or Doula or “Human Resources Officer” or “HR worker” or “Volunteer” Or “Helping professional” or “medical rescuer” or physiotherapist or “workers in hospital” or “palliative care” or teacher* or pedagogue* or fireman or vet or educator* or psychiatrist* or “medical doctor” **(Topic)**.

**FIGURE 1:**
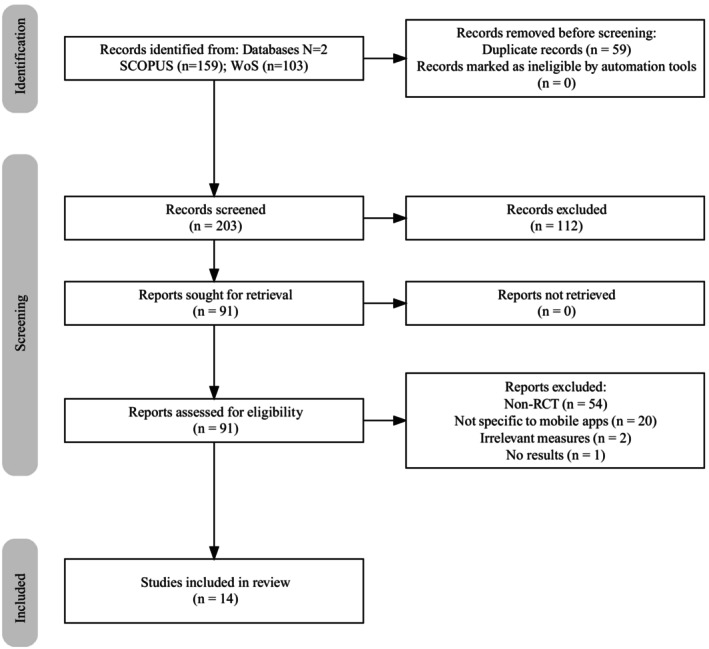
PRISMA flow diagram.

The articles were inputted into Zotero (version 6.0.30), a free open‐source software for managing bibliographic data. The titles and abstracts were then screened to remove irrelevant studies and duplicates. Fifteen studies involving mobile applications fully met the inclusion criteria (Figure 1).

### 2.4 Data Synthesis for the Meta‐Analysis

We conducted a meta‐analysis using Meta‐Essentials (Van Rhee et al. 2015) on 10 studies with comparable scales: Maslach Burnout Inventory (MBI) or Maslach Burnout Inventory–Human Services Survey (MBI‐HSS; Maslach et al. 1997), and the Oldenburg Burnout Inventory (OLBI; Demerouti et al. 2001). The analysis was performed on 10 studies with emotional exhaustion and depersonalization scales, and 9 studies with the personal accomplishment scale. We used a random‐effects model and estimated effect size in Hedge's *g*. Heterogeneity was assessed using Q statistics and I^2^ index. To check whether any single study influenced findings, we performed a leave‐one‐out sensitivity analysis (Cumpston et al. 2019). This involved iteratively excluding each study one at a time and recalculating the overall effect size to determine whether any single study disproportionately influenced the results. This approach ensures that the meta‐analytic estimates are not affected by outliers or methodological inconsistencies in individual studies. To maintain transparency and avoid selective reporting bias, we report results both before and after sensitivity analysis.

Lambert et al. (2020) used interquartile range and median to report results. We evaluated the skewness of the data and estimated sample means using the method suggested by Luo et al. (2016) and the standard deviations proposed by Wan et al. (2014).

McGinness et al. (2022) used the OLBI, from which the disengagement scale was mapped to depersonalization, and the exhaustion scale was aligned with emotional exhaustion.

Publication bias was tested using Egger's linear regression method and funnel plots (Van Rhee et al. 2015) following a sensitivity analysis on results exhibiting low heterogeneity.

## 3 Results

### 3.1 Study Characteristics

Three of the 14 articles were quasi‐experimental, and 11 were RCTs. All were published in peer‐reviewed journals. Most participants were female, ranging from 38% to 100%. Ages ranged from 27 to 46 years. Research mainly focused on healthcare professionals (*n* = 9), nurses (*n* = 3), and other helping professionals (*n* = 2). The studies contained between 20 and 2182 participants. Nine of them were conducted in the USA and Canada. For a description of the studies and intervention characteristics, see Table 2.

**TABLE 2:** Study characteristics.

Author, year (Country)	Design	Participants (Number, female ratio, occupation)	Mean age	Measure	Intervention		Measurements time points
					Type, duration, comparison	Description	
Kirykowicz et al. (2023) (South Africa)	RCT	E = 16, C = 17 58.8% Doctors and medical personnel	33.4	CBI	Type: COVID Coach app Duration: 1 month Comparison: waitlist, wait‐to‐treat	Self‐management app with relaxation and mindfulness audio exercises, mental health educational content, and mood tracking	Pre, post
Russell and Smyth (2023) (Ireland)	RCT	E = 29, C = 15 100% Special educators	33.45	CBI	Type: ‘Smiling Mind’ app Duration: 10 days Comparison: Day One’, a note‐taking app	Mindfulness app with guided meditations	Pre, post f/u: 2 weeks
Wylde et al. (2017) (USA)	Quasi‐experimental	E = 46, C = 49 92% Novice Pediatric Nurses	N/a	ProQOL, CFST	Type: Headspace app Duration: 4 weeks Comparison: traditionally delivered mindfulness intervention	Mindfulness app with brief meditation practices and psychoeducational content	Pre, post
Jakel et al. (2016) (USA)	Quasi‐experimental	E = 16, C = 9 76% Oncology nurses	N/a	ProQOL	Type: Provider app Duration: 6 weeks Comparison: waitlist	Resilience app with psychoeducation about CF, physical exercises, video, and daily affirmations	Pre, post
McGinness et al. (2022) (USA)	Quasi‐experimental	E = 20, C = 21, E = 90%, C = 67 Pediatric residents	28.8	OLBI, PWLS	Type: Gratitude and 3 good things app Duration: 4 weeks Comparison: waitlist	Journaling app with three things to be grateful for and three good things	Pre, post f/u: 1 month, 6 months
Fiol‐DeRoque et al. (2021) (Spain)	RCT	E = 248, C = 234 83.2% Health Care Workers	42	MBI‐HSS	Type: PsyCovidApp Duration: 2 weeks Comparison: control app with general recommendations about mental health care	Psychoeducational app, based on cognitive‐behavioral therapy and mindfulness with written, audio and visual content	Pre, post
Lambert et al. (2020) (USA)	RCT	E = 13, C = 12 44% Emergency medicine personnel	38	MBI‐HSS	Type: Stress free meditation for Healers app Duration: 90 days Comparison: waitlist	Meditation app with twelve guided meditations	Pre, post f/u: 6 months
Mistretta et al. (2018) (USA)	RCT	E = 22, E2 = 23, C = 15 86.7% Employees of the healthcare institution	46.0	MBI‐HSS	Type: Resiliency‐based app Duration: 6 week Comparison: in‐person mindfulness‐based resilience training, waitlist	Resilience app with focus on sleep tracking and well‐being awareness, including topics on positivity, energy and focus, productivity and sleep	Pre, post f/u: 3 months
Boucher et al. (2023) (Canada)	RCT	E = 142, C = 146 85.4% Health care workers	41	MBI	Type: Down dog app Duration: 12 week Comparison: waitlist	Body weight interval training, yoga, barre, and running	Pre, post
Monfries et al. (2022) (Canada)	RCT	E = 8, C = 12 81.6% Health care workers from emergency department	N/a	MBI	Type: Headversity app Duration: 3 months, Comparison: waitlist	Resilience app with structured curriculum and training modules on: mental fitness and health, mindfulness, energy management, hardiness and self‐expertise	Pre, post
Pandya (2019) (USA)	RCT	E = 45, C = 40 38% Chaplains working with elderly	42.5	MBI	Type: Meditation app with videos and audio sessions Duration: 1 year Comparison: leisure app	Meditation app with video and audio guided content	Pre, post
Pratt et al. (2023) (USA)	RCT	E = 33, C = 69 94.1% Nurses	26.5	MBI	Type: Lift mobile Duration: 1 month Comparison: waitlist	Mindfulness app with video and audio guided content	Pre, post
Taylor et al. (2022) (UK)	RCT	E = 1095, C = 1087 41.52% Healthcare helping professionals	40.42	MBI	Type: Headspace app Duration: 1.5 months Comparison: Moodzone app and platform	Mindfulness app with brief meditation practices and psychoeducational content	Pre, post f/u: 4.5 months
Xu et al. (2021) (Australia)	RCT	E = 74, C = 74 78% Emergency department staff	37	MBI	Type: Headspace app Duration: 4 weeks Comparison: waitlist, wait‐to‐treat	Mindfulness app with brief meditation practices and psychoeducational content	Pre, post f/u: 3 months

Abbreviations: BO, burnout; C, control; CBI, copenhagen burnout inventory; CF, compassion fatigue; CFST, the compassion fatigue self‐test; CS, compassion satisfaction; E, experimental; f/u, follow‐up; MBI, Maslach Burnout Inventory; MBI‐HSS, Maslach Burnout Inventory‐Human Services Survey; OLBI, Oldenburg Burnout Inventory; ProQOL, professional quality of life; PWLS, physician work life study; RCT, randomized controlled trials.

Mobile applications were used in all the studies, either as a control or waitlist condition, or both. The interventions were designed to reduce burnout and compassion fatigue and increase compassion satisfaction. The content of interventions included mindfulness and meditation programs (*n* = 7), resilience‐based interventions (*n* = 3), and other methods such as body training, psychoeducation, self‐management, and journaling (*n* = 1 each).

The programs ran from 10 days to 1 year. Most of the studies (*n* = 8) had a waitlist or a wait‐to‐treat group (the waitlist group becomes the control condition after the first group has finished the intervention). Burnout was measured using Maslach Burnout Inventory (MBI; *n* = 6) or Maslach Burnout Inventory‐Human Services Survey (MBI‐HSS; *n* = 3; Maslach et al. 1997), Copenhagen Burnout Inventory (CBI; *n* = 2; Kristensen et al. 2005) or other (*n* = 2); compassion fatigue and compassion satisfaction were measured using the Professional Quality of Life Scale (ProQOL; *n* = 2; Stamm 2010). Two studies measured similar constructs using two different questionnaires. Six studies had a follow‐up ranging from 2 weeks to 6 months.

### 3.2 Risk‐Of‐Bias Assessment

**FIGURE 2:**
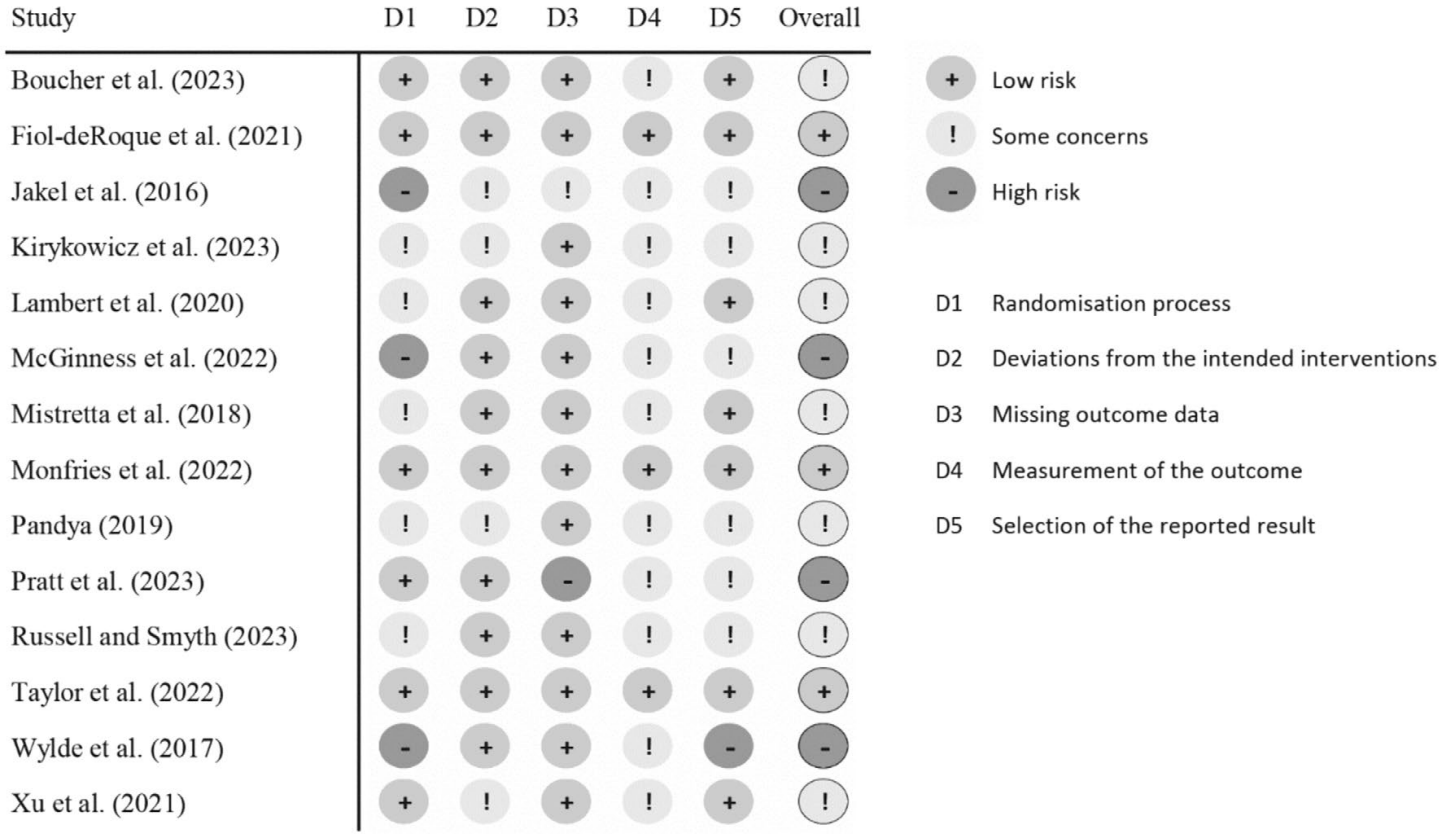
Cochrane risk‐of‐bias evaluation for randomized controlled trials (RoB 2.0) (Sterne et al. 2019).

Risk of bias was evaluated by two independent researchers (D.D. and L.K.) and discussed with the auditor (J.H.) using Cochrane's Risk of Bias 2.0 (RoB 2.0; Sterne et al. 2019) for RCTs (Figure 2). Overall, there were “some concerns” of risk of bias, with three studies assessed as “low risk” (Fiol‐DeRoque et al. 2021; Monfries et al. 2022; Taylor et al. 2022), seven raising “some concerns” (Boucher et al. 2023; Kirykowicz et al. 2023; Lambert et al. 2020; Mistretta et al. 2018; Pandya 2019; Russell and Smyth 2023; Xu et al. 2021) and four at “high risk” (Jakel et al. 2016; McGinness et al. 2022; Pratt et al. 2023; Wylde et al. 2017).

### 3.3 Systematic Review: Compassion Fatigue, Compassion Satisfaction, and Burnout

Compassion fatigue and compassion satisfaction were measured directly in two quasi‐experimental studies using ProQOL (Stamm 2010). A study by Jakel et al. (2016) reported a slight decrease in secondary traumatic stress in the experimental group, but none of the group differences were considered significant in the three ProQOL dimensions. Wylde et al. (2017) compared traditional and smartphone mindfulness interventions. The smartphone group showed marginally higher compassion satisfaction and lower burnout scores than the active control group, but the differences were not significant. In the smartphone group, compassion fatigue was lower in nurses not suffering from clinical post‐traumatic stress disorder compared to the active control group. Such symptoms were associated with higher burnout and compassion fatigue, supporting the link to worse outcomes.

Two studies (Kirykowicz et al. 2023; Russell and Smyth 2023) that were not included in the meta‐analysis that used the CBI scale (Kristensen et al. 2005) did not report group differences for the burnout measurements.

Other studies included in the meta‐analysis showed that mobile interventions had various effects on burnout. Several studies found there were significant reductions in burnout facets, such as emotional exhaustion and depersonalization. For instance, Boucher et al. (2023) found significant treatment effects for these two facets, while Monfries et al. (2022) and McGinness et al. (2022) reported significant reductions in emotional exhaustion, with the latter suggesting potential for sustained intervention effects. Lambert et al. (2020) found that the intervention group experienced significant improvements in feelings of personal accomplishment. Xu et al. (2021) reported statistically significant improvements in all three components of burnout (personal accomplishment, emotional exhaustion, and depersonalization) from pre‐intervention to 3 months later, with small effect sizes. Similarly, Pandya (2019) indicated that chaplains who used the M‐App exhibited less emotional exhaustion and depersonalization and higher personal achievement and resilience in a 12‐month longitudinal study.

However, some studies, such as Mistretta et al. (2018), Fiol‐DeRoque et al. (2021), and Taylor et al. (2022), reported small non‐significant changes. Additionally, Pratt et al. (2023) found that Maslach Burnout Inventory depersonalization scores fell more in the control than in the intervention arm.

### 3.4 Meta‐Analysis: Burnout

#### 3.4.1 Personal Accomplishment

The combined effect size for change in the Personal Accomplishment scale was significant (*p* = 0.044), Hedge's *g* = 0.52. Heterogeneity was detected in the studies (*Q* = 94, *p*_Q_ < 0.000), and confirmed by *I* ^2^ = 91%, suggesting that the populations are different (Table 3 and Figure 3).

**TABLE 3:** Effect sizes for interventions to improve personal accomplishment in helping professionals.

#	Study name	Hedges' *g*	CI lower limit	CI upper limit	Weight
1	Boucher et al. (2023)	0.18	−0.06	0.42	13.24%
2	Fiol‐deRoque et al. (2021)	0.12	−0.07	0.31	13.55%
3	Lambert et al. (2020)	0.48	−0.32	1.31	8.53%
4	Mistretta et al. (2018)	0.17	−0.42	0.76	10.33%
5	Monfries et al. (2022)	0.55	−0.36	1.51	7.67%
6	Pandya (2019)	3.05	2.45	3.71	9.87%
7	Pratt et al. (2023)	0.17	−0.27	0.62	11.60%
8	Taylor et al. (2022)	0.00	−0.10	0.11	13.90%
9	Xu et al. (2021)	0.47	−0.01	0.96	11.29%

**FIGURE 3:**
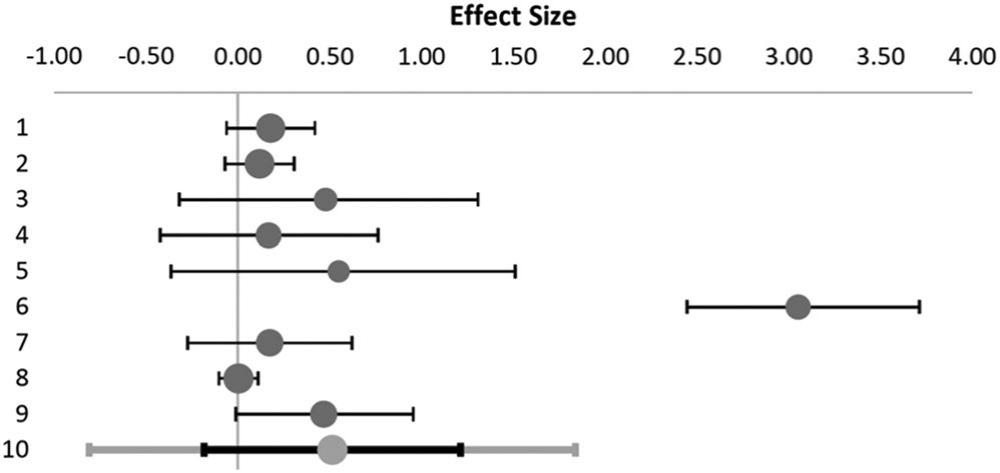
Meta‐analytical results of effect sizes for personal accomplishment in Hedges' *g* (95% CI). The relative size of the gray bullets represents the study's weight in the meta‐analytic result. Bullet №10: weighted average effect (combined effect size), black—confidence interval, gray—prediction interval.

#### 3.4.2 Emotional Exhaustion

The combined effect size for change in Emotional Exhaustion scale was not significant (*p* = 0.063), Hedges' *g* = 0.67. Heterogeneity was detected in the studies (*Q* = 141.53, *p*_Q_ < 0.000), and confirmed by *I* ^2^ = 94%, suggesting that the populations are different.

#### 3.4.3 Depersonalization

The combined effect size for change in the Depersonalization scale was non‐significant (*p* = 0.134), Hedge's *g* = 0.47. Heterogeneity was detected in the studies (*Q* = 137.27, *p*_Q_ < 0.000), and confirmed by *I* ^2^ = 93%, suggesting that studied populations are different.

#### 3.4.4 Sensitivity Analysis

The sensitivity analysis identified one study (Pandya 2019) driving the results of the meta‐analysis; its exclusion radically improved the homogeneity of all three scales. No other study had a significant impact on the results of our research and had a similar weight in the results.

**FIGURE 4:**
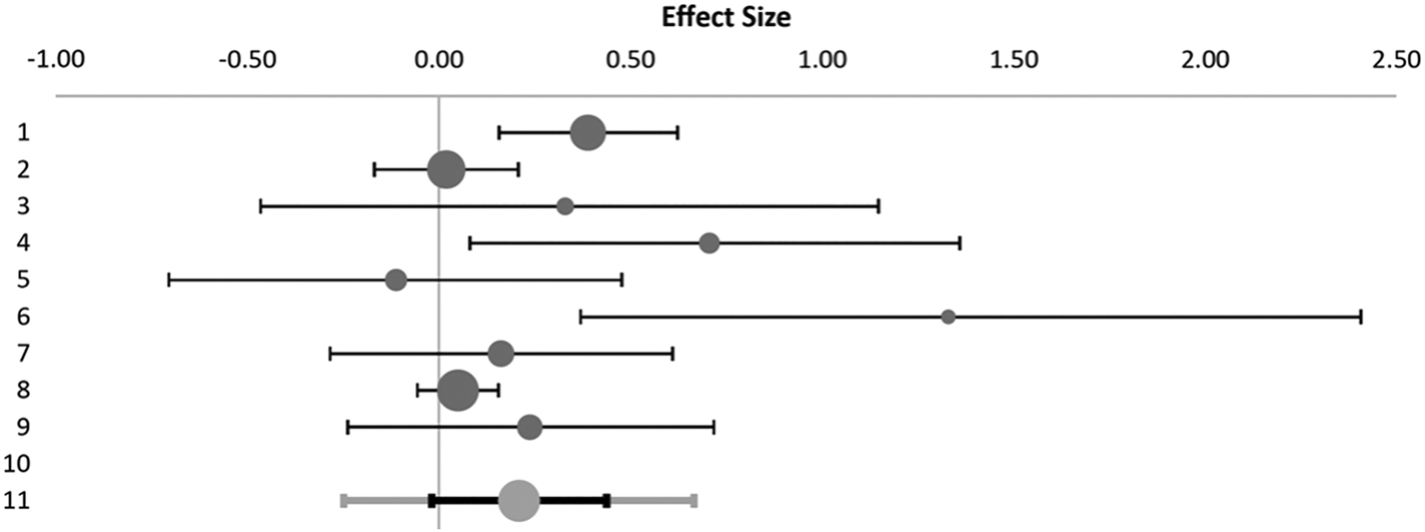
Meta‐analysis results of effect sizes for emotional exhaustion in Hedges' *g* (95% CI) after the sensitivity analysis.

After excluding the outlier, the decrease in Emotional Exhaustion was significant (*p* = 0.017), Hedge's *g* = 0.21, *Q* = 18.79, *p*_Q_ = 0.016, *I* ^2^ = 57% (moderate heterogeneity concerns) (Cumpston et al. 2019). Egger's regression and the funnel plot showed a low likelihood of publication bias (*p* = 0.081). See Table 4 and Figure 4.

**TABLE 4:** Meta‐analysis model for the emotional exhaustion scale after the sensitivity analysis.

Model	Random effects model
Confidence level	95%
Combined effect size	
Hedges' *g*	0.21
Standard error	0.10
CI lower limit	−0.02
CI upper limit	0.44
PI lower limit	−0.25
PI upper limit	0.67
Number of subjects	2395
Number of studies	9
Heterogeneity	
*Q*	18.79
*p*_Q_	0.016
*I* ^2^	57.43%
*T* ^2^	0.03
*T*	0.17

Personal accomplishment remained significant (*p* = 0.018), with Hedge's *g* = 0.10, *Q* = 7.79, *p*_Q_ = 0.352, *I* ^2^ = 10% (unimportant heterogeneity concerns). Egger's regression and the funnel plot showed that publication bias was likely (*p* = 0.003).

Depersonalization remained non‐significant (*p* = 0.193), Hedge's *g* = 0.09, *Q* = 19.70, *p*_Q_ = 0.012, *I* ^2^ = 59% (moderate heterogeneity concerns). Egger's regression and the funnel plot showed a low likelihood of publication bias (*p* = 0.792).

#### 3.4.5 Moderator and Subgroup Analysis by Intervention Length and Program Type

We assessed the moderation effect of the intervention length on the results of the meta‐analysis. None of the scales were significant after the sensitivity analysis, but all the studies had a positive slope (Table 5).

**TABLE 5:** Regression of intervention length as a moderator of effect size.

Moderator	*B*	SE	CI LL	CI UL	*Β*	*Z*	*p*
Depersonalization	0.03	0.02	−0.03	0.08	0.36	1.22	0.224
Personal accomplishment	0.01	0.02	0.03	0.05	0.24	0.57	0.572
Emotional exhaustion	0.04	0.02	0.01	0.09	0.53	1.80	0.071

We performed a subgroup analysis by program type, comparing interventions such as mindfulness, resilience training, and others. The results were insignificant across all three burnout subscales, indicating no differences between the groups based on the intervention type.

## 4 Discussion

### 4.1 Overview

In the current research study, we analyzed 14 studies as part of the systematic review, and 10 articles were included in the meta‐analysis. Our work is the first to synthesize data across diverse healthcare professionals and interventions targeting burnout, compassion fatigue, and compassion satisfaction delivered exclusively via mobile apps. By addressing these gaps, our findings provide novel evidence for low‐burden mobile interventions as tools to mitigate occupational stress and improve professional quality of life. We followed the PRISMA guidelines (Sterne et al. 2019) and utilized the PICO framework (Schardt et al. 2007) to synthesize results from the RCT and quasi‐experimental studies.

### 4.2 Compassion Fatigue, Compassion Satisfaction, Burnout: Systematic Review

Nine of the 14 articles were conducted in the USA and Canada. This trend aligns with Sweileh (2020), who reported that a plurality of articles published on burnout and compassion fatigue (*n* = 1292; 29.3%) was published in the USA. This could indicate that researchers from low‐ and middle‐income countries are less focused on burnout and compassion fatigue and that interventional strategies are not used so much in these countries or that this area attracts less published research. However, a recent meta‐analysis showed that nurse burnout in the Southeast Asia and the Pacific region has the highest prevalence rate of the six geographical regions (Woo et al. 2020). Nonetheless, there is a need for more internationally available mobile applications as well as more research studies with international samples of participants.

The concentration of research in high‐income countries may also reflect disparities in healthcare resources and infrastructure, which can affect the availability and implementation of interventions. For example, healthcare systems in low‐ and middle‐income countries may face challenges such as lower levels of funding, workforce shortages, and high patient‐to‐provider ratios, which can exacerbate burnout and compassion fatigue among healthcare professionals (WHO guidelines on mental health at work 2022). Addressing these issues requires both more research and tailored interventions that consider the contexts and needs of healthcare providers in different regions.

The participants in the analyzed studies were mostly women. This finding is supported by the Global Gender Gap Report, which indicates that women comprise over 70% of employees in health care and social assistance sectors (WEF 2021).

The most common interventions were mindfulness, meditation, and resilience‐based training. While mindfulness and resilience apps had mixed results, most studies that used meditation showed improvements in burnout domains. Two studies that directly evaluated compassion fatigue and compassion satisfaction using ProQOL (Stamm 2010) showed no group differences. The convenience sample, small group size, brief intervention program, lack of adherence rates, and frequency of intervention method may have affected the results (Jakel et al. 2016; Wylde et al. 2017).

Our search focused on trials from 2010 to 2024, but the earliest study was from 2016, and most were conducted recently, indicating that mobile application usage has increased in recent years. This is supported by an increase in research publications on health‐promoting mobile apps, from fewer than five studies in 2012 to more than 50 in 2018 (Iribarren et al. 2021). Additionally, nine of the studies in our research were performed after 2021, possibly reflecting heightened interest in mobile app interventions for mitigating burnout and compassion fatigue during and after the COVID‐19 pandemic. Despite limited evidence on the efficacy of mobile interventions, there was an increase in such studies during the COVID‐19 pandemic (Rauschenberg et al. 2021).

### 4.3 Burnout: Meta‐Analysis

The meta‐analysis results revealed a significant effect observed for personal accomplishment both before (medium effect size) and after the sensitivity analysis (small effect size). This implies stable interventions' effects with enhanced positive outcomes. The combined effect size for emotional exhaustion was significant with a small magnitude only after the sensitivity analysis, showing mixed efficacy. Considering the large effect size of the original outlier study that was removed after the sensitivity analysis (Pandya 2019), such findings imply that the combined effect of interventions on emotional exhaustion was positive, but it requires further investigation. While some studies reported positive outcomes (Boucher et al. 2023; Pandya 2019; Xu et al. 2021), overall changes in depersonalization remained non‐significant.

We can hypothesize that while mobile apps may not always alleviate negative burnout symptoms, they enhance feelings of professional efficacy and achievement and foster a sense of accomplishment. This outcome aligns with the nature of many interventions in the review, such as mindfulness and resilience‐based programs, which are often designed to promote “positive” psychological states rather than merely reduce negative symptoms (Allen et al. 2021).

Additionally, the measurement instruments may have been more sensitive to this positive effect. Contrary to our findings, another meta‐analysis focused on burnout in clinical nurses found no increase in personal accomplishment (Lee and Cha 2023). This discrepancy could also be attributed to differences in the studied populations, as our inclusion criteria for healthcare professionals were broader, or the means of delivery—we focused solely on mobile apps.

### 4.4 Quality of Evidence

The majority of studies raised some concerns, which partly affect the reliability of the findings. Three studies had a low risk of bias (Fiol‐DeRoque et al. 2021; Monfries et al. 2022; Taylor et al. 2022), and four studies had a high risk (Jakel et al. 2016; McGinness et al. 2022; Pratt et al. 2023; Wylde et al. 2017). The higher level of bias is primarily due to issues such as lack of blinding, measurement of the outcome data, and selective reporting.

The high levels of heterogeneity detected in the meta‐analysis for emotional exhaustion (*I* ^2^ = 94%), depersonalization (*I* ^2^ = 93%) and personal accomplishment (*I* ^2^ = 91%) suggest substantial variability between studies, which drastically decreased after a sensitivity analysis and the exclusion of one study (Pandya 2019) that was driving the results of our meta‐analysis. While some of the variability can be attributed to differences in intervention type, population, and the measurement tools used in the studies, the exclusion of this study resulted in greater homogeneity in the samples, indicating that our findings may be more generalizable.

We considered that Pandya (2019) shows how adherence to intervention and longitudinal design might influence the results. While we were unable to find comparable studies of a similar duration (up to 1 year), additional analysis revealed that intervention length acted as a moderator. Even after excluding the outlier, all three scales showed a positive slope, though none were significant. Greater attention should be paid to intervention length in future research and meta‐analyses. To our knowledge, previous studies have focused mainly on the novelty factor, which diminishes significantly after 8 weeks, falling to less than 1% of the variance (Chwo et al. 2018). This suggests that studies shorter than 8 weeks may have positively skewed the results owing to this novelty effect. At the same time, we did not find any studies specifically assessing intervention length as a moderator on compassion fatigue, compassion satisfaction, and burnout.

Unfortunately, the insufficient number of studies reporting adherence rates meant we could not investigate it as a moderator. For example, Xu et al. (2021) provide very limited information, noting that only half of the participants reported continuous app usage. Meanwhile, Russell and Smyth (2023) examined the relationship between adherence and burnout subscales but found no significant correlations. Most studies did not explicitly mention adherence, which could be a design limitation. Gathering such information centrally and linking it to participants' IDs could prove difficult, possibly because of data anonymization. Researchers may need to invest additional effort into developing methods to foster and measure adherence to their interventions, adopting frameworks for adherence measurement that include multi‐dimensional metrics (e.g., session attendance, intervention duration, user engagement) to enable cross‐study comparisons and strengthen interpretations of intervention efficacy (Giovanazzi et al. 2022).

### 4.5 Implications and Future Research

Despite the promising results on burnout, our study did not find a single replicated method that effectively addressed overall professional quality of life, including both compassion fatigue and compassion satisfaction. Many interventions are linked to meditation and mindfulness practices and resilience training, but there is a lack of consistency in the methods and apps used. This makes it difficult to determine the most effective approaches, which was supported by insignificant results in the subgroup analysis by program type. Future research should focus on standardized approaches and replication of findings using the same apps rather than a broad range of techniques. This should help to identify the most effective methods for reducing burnout and compassion fatigue and enhancing compassion satisfaction. A key recommendation from the studies reviewed is to implement more rigorous methodologies, such as blinding both data analysts and participants regarding the intervention received and the outcomes measured, to improve the reliability of the findings.

Additionally, most research focuses primarily on burnout, which may be due to the inconsistent reliability and validity of current compassion fatigue measurement tools. A recent systematic meta‐analysis of the widely used ProQOL scale (Stamm 2010) revealed that secondary traumatic stress and burnout dimensions need revision and have loading problems (Hotchkiss and Wong 2022). At the same time, the MBI tool (Maslach et al. 1997) and the burnout definition used in its manual have been criticized on both conceptual and psychometric grounds and yet are used in about 90% of all scientific publications on burnout (Schaufeli et al. 2023), a finding that is supported by our systematic review results. There is a need for better measurement tools that are reliable, valid, and applicable across different cultures. The lack of studies is reflected in a recent systematic review (Moore et al. 2024), which investigated mobile interventions for wellbeing in nurses but found no relevant studies specifically addressing compassion fatigue and compassion satisfaction.

Currently, most research is done on healthcare professionals; it is essential to broaden the scope to include other helping professionals. Evidence suggests that individuals working in various people‐facing professions, not just healthcare, are susceptible to compassion fatigue, secondary traumatic stress, and burnout (Bride 2007; Sprang et al. 2007).

## 5 Linking Evidence to Action

- Interventions via mobile applications enhance personal accomplishment among healthcare professionals, while their effectiveness in reducing emotional exhaustion is mixed, and results for depersonalization are not significant.
- Mobile applications with interventions targeting burnout and compassion fatigue and satisfaction primarily focus on mindfulness, meditation, and resilience training.
- Evidence for high‐quality interventions targeting compassion fatigue and satisfaction remains very limited, with insignificant results, highlighting the need for further research in the form of RCTs.
- Adherence to mobile interventions should be measured and reported consistently to ensure reliable evaluation of their effectiveness.
- Current research predominantly focuses on healthcare professionals, despite evidence suggesting that a broader range of healthcare professionals suffers from compassion fatigue and burnout.

## 6 Conclusion

This study provides the first systematic synthesis of interventions via mobile apps targeting burnout, compassion fatigue, and compassion satisfaction across diverse healthcare professionals. Mindfulness, resilience, and other intervention programs delivered through mobile apps have shown significant benefits for improving burnout, particularly in personal accomplishment, offering low‐burden strategies to support professional efficacy. Interventions demonstrated mixed but promising efficacy in reducing emotional exhaustion, while results for depersonalization were not significant, highlighting the need for interventions tailored to specific burnout dimensions.

Studies focusing specifically on compassion fatigue did not show significant group differences, suggesting that mobile interventions have not consistently been effective in addressing compassion fatigue and compassion satisfaction, underscoring a critical gap in current research. The limited number of studies on this topic may have affected the results, further emphasizing the need for trials. However, the observed positive impact on personal accomplishment may potentially benefit compassion satisfaction, indicating that interventions via mobile apps can enhance job satisfaction and a sense of achievement, even without directly reducing compassion fatigue.

## Ethics Statement

All procedures performed in studies involving human participants were by the ethical standards of the institutional and/or national research committee and with the 1964 Helsinki Declaration and its later amendments or comparable ethical standards. The study's protocol was approved by the Ethical committee of the Faculty of Social and Economic Sciences at Comenius University Bratislava FSEV 1647/‐4/2022/SD‐CIII/1.

## Consent

Informed consent was obtained from all individual participants included in the study.

## Conflicts of Interest

The authors declare no conflicts of interest.

## Data Availability

The datasets generated and/or analyzed during the current study are not publicly available due to the ethical reasons but are available from the corresponding author on reasonable request.

## Appendix 1 List of Helping Professionals

1. Healthcare providers: nurses, doctors, surgeons, physicians, home health workers, hospice/palliative care workers, medical residents, nursing students, public health workers, burn care providers, pediatric healthcare providers, oncology social workers, pharmacists, dentists, and doulas.
2. Mental health professionals: psychologists, psychotherapists/therapists, trauma counselors, grief counselors, addiction counselors, mental health workers, psychiatrists.
3. Social and community services: social workers, child welfare/protective services workers, domestic violence advocates, shelter workers, human resources officers, HR workers, humanitarian aid workers, and volunteers (e.g., disaster responders)
4. Emergency and crisis responders: paramedics/medical rescuers, firefighters (firemen/firewomen), search and rescue personnel, disaster mental health responders, disaster relief workers.
5. Law and justice professionals: police officers, lawyers, judges, attorneys, forensic interviewers, legal/court personnel.
6. Spiritual and religious support: clergy, pastors, priests, nuns, monks, missionaries.
7. Animal care professionals: veterinarians, animal shelter staff.
8. Educational professionals: teachers, pedagogues, special/therapeutic educators, trainers, coaches, mentors.
9. Therapy and rehabilitation: occupational therapists, physical therapists/physiotherapists.
